# Durable Complete Remission With Combination of Stereotactic Body Radiation Therapy (SBRT) and Talimogene Laherparepvec (TVEC) Followed by Ipilimumab in Refractory Metastatic Melanoma

**DOI:** 10.7759/cureus.29573

**Published:** 2022-09-25

**Authors:** Zachary Wolfe, Aaron Blackham, Alyson McIntosh, Angela Miller, Hina Sheikh, Suresh Nair

**Affiliations:** 1 Hematology and Medical Oncology, Lehigh Valley Topper Cancer Institute, Allentown, USA; 2 Surgical Oncology, Lehigh Valley Topper Cancer Institute, Allentown, USA; 3 Radiation Oncology, Lehigh Valley Cancer Institute, Allentown, USA; 4 Pathology, Health Network Laboratories, Allentown, USA; 5 Hematology and Oncology, Lehigh Valley Cancer Institute, Allentown, USA

**Keywords:** targeted therapeutics, oncolytic viruses, radiation therapy, immunotherapy, melanoma

## Abstract

Metastatic melanoma refractory to checkpoint inhibitors is a challenging clinical scenario. We present the case of a patient who was refractory to standard of care but was able to achieve a durable complete remission with the combination of stereotactic body radiation therapy (SBRT), talimogene laherparepvec (TVEC), and ipilimumab.

## Introduction

While immunotherapy has dramatically changed the landscape of melanoma treatment, not all patients will respond to checkpoint inhibitors. Data have suggested that combining checkpoint inhibitors with other modalities of therapy may increase the response rate and perhaps convert a non-responder to a responder [1-3]. Checkpoint inhibitors have been combined with radiation therapy in case reports and case series and with talimogene laherparepvec (TVEC) in randomized trials [1-4]. We present a case of a heavily pretreated patient with metastatic melanoma who failed to respond to TVEC plus ipilimumab but responded dramatically to the combination of TVEC plus stereotactic body radiation therapy (SBRT) followed by ipilimumab.

## Case presentation

**Figure 1:**
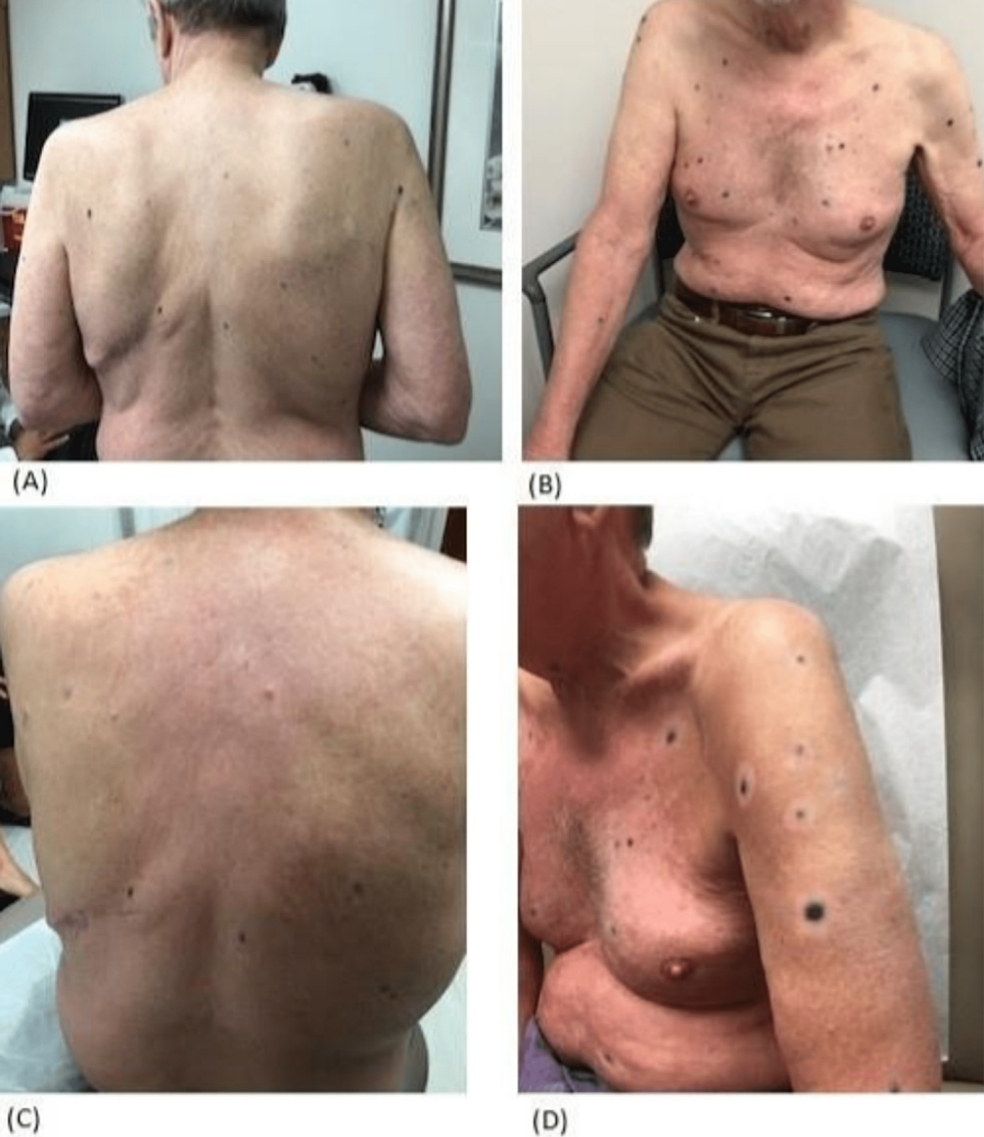
Cutaneous metastases demonstrating treatment response. (A-B) Cutaneous lesions prior to combined treatment with SBRT, TVEC, and ipilimumab. (C-D) Cutaneous lesions after treatment with SBRT and TVEC followed by ipilimumab. SBRT, stereotactic body radiation therapy; TVEC, talimogene laherparepvec

A 66-year-old male patient with a five-year history of BRAF V600E positive melanoma was seen for progressive metastatic disease. He was initially diagnosed in 2012 with a T2a (1.2mm, non-ulcerated) superficial spreading cutaneous melanoma on his back five years prior which was treated with local excision alone. He developed local recurrence 17 months later. Wide local excision and sentinel lymph node biopsy (SLNB) was done with pathology revealing a locally recurrent melanoma extending to a depth of 11 mm with associated microsatellite lesions. The sentinel lymph node (SLN) was negative. He was treated with adjuvant peginterferon alfa-2b. Twelve months later he developed three cutaneous metastases for which he received TVEC plus ipilimumab in a clinical trial but developed progressive cutaneous metastases. Treatment was switched to pembrolizumab and he had a complete response to his cutaneous lesions. A positron emission tomography-computed tomography (PET-CT) scan after one year of therapy showed no residual cutaneous activity but did reveal one FDG (fluorodeoxyglucose)-avid left axillary lymph node which was treated with SBRT. He had a complete response (CR) after SBRT for five months until he presented with a recurrent metastatic lesion on his back. At this point, nivolumab was started. After three months, he developed further cutaneous metastases with a total of eight lesions ranging in size from 4 to 7 mm. Therefore, dabrafenib and trametinib were started and although he had a good response, he developed autoimmune side effects including grade 3 trigeminal neuralgia, grade 2 pyrexia, chills, and shortness of breath. Dabrafenib and trametinib were discontinued. Shortly thereafter, more than 20 new cutaneous metastases appeared (Figure 1A-B).

**Figure 2:**
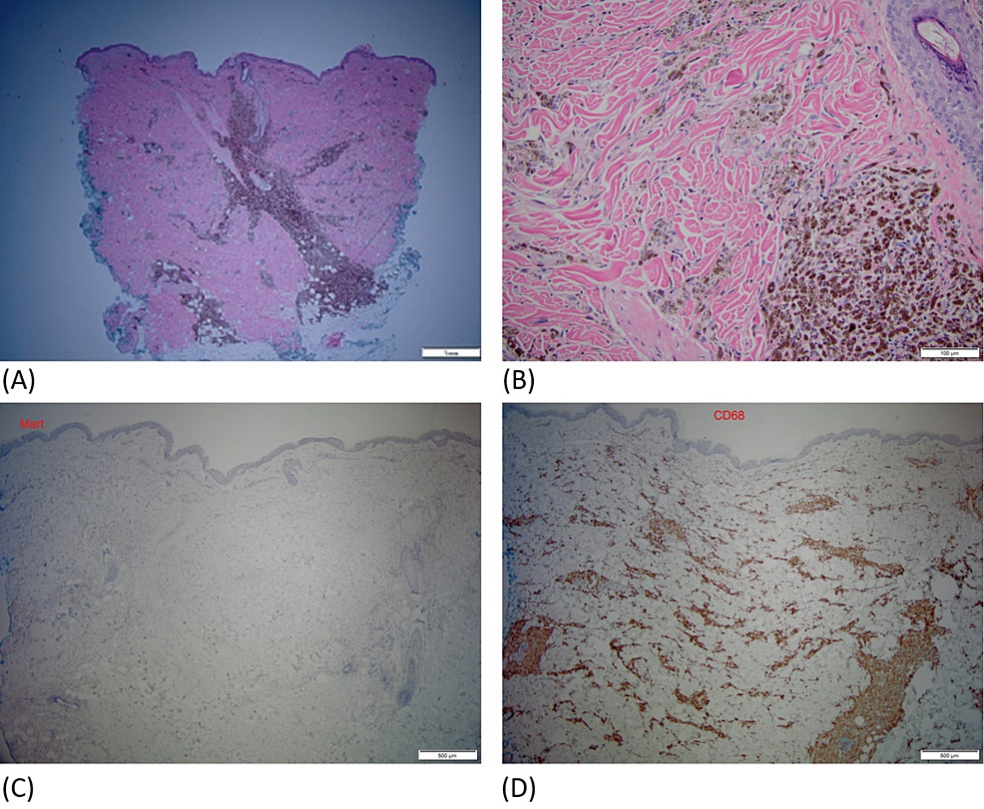
Pathology. (A) H&E-stained section showing a heavy infiltrate of pigmented cells in the dermis in perivascular and perifollicular distribution, magnification 2x. (B) Their bland cytology and coarse melanin granules, magnification 20x. (C) Immunostain for Mart (melanoma marker) is negative, magnification 4x (note: endogenous melanin pigment has been removed for immunostain study). (D) Immunostain for CD68 (histiocyte marker) is diffusely positive in the same area, confirming that pigmented cells are melanophages (melanin-laded macrophages), magnification 4x.

Given his limited options, we recommended therapy with a combination of ipilimumab (3 mg/kg every 3 weeks) and TVEC. He received two treatments of TVEC (0.5 mL) to eight cutaneous lesions, after which a PET scan showed a new adrenal metastasis. This was treated with SBRT (5 Gy over five fractions) followed by low-dose ipilimumab (3 mg/kg every 3 weeks for four doses). The patient developed recurrent episodes of arthralgias and myalgias requiring brief courses of corticosteroids. He also had a recurrence of trigeminal neuralgia which again resolved with corticosteroids. New metastatic lesions continued to appear until after the second cycle of ipilimumab. Four months after completing ipilimumab, the hyperpigmented lesions began to fade and a vitiliginous halo had developed around every lesion (Figure 1c-d). He underwent a punch biopsy of four different cutaneous lesions, including one that had been previously treated with TVEC, one that had not been treated, one that had appeared after treatment with TVEC, and one that appeared during treatment with ipilimumab. In all lesions, pathology showed no residual malignant melanoma but only melanin-laden macrophages indicating a complete pathologic response to therapy (Figure 2A-D).

Repeat PET-CT showed no FDG-avid lesions. He remains in complete remission four years after completing ipilimumab.

## Discussion

Checkpoint inhibition has revolutionized the treatment of melanoma, but not all patients will respond and relapses are common. This patient had relapsed progressive melanoma after four lines of therapy. However, he was able to achieve a durable complete remission after receiving a second course of TVEC followed immediately by SBRT and ipilimumab, despite being refractory to TVEC and ipilimumab previously. This case suggests that refractoriness to immunotherapy may be overcome by the addition of local therapies (TVEC or SBRT). Both TVEC and SBRT have been postulated to be able to act synergistically with checkpoint inhibitors.

The TVEC has been studied in combination with ipilimumab and the combination showed improved response rates over ipilimumab alone (39% vs. 18% odds ratio, OR 2.9; confidence interval, CI 1.5-5.5, p=0.002) in a randomized phase II trial [2]. The addition of TVEC to pembrolizumab did not improve progression free survival (PFS) or overall survival (OS) in a large phase III trial [5]. However, this trial was conducted in anti-PD-1 (programmed cell death protein 1) therapy naïve patients, not refractory patients. Little is known about the role of TVEC in this patient population, but other cases in addition to our own, suggest that TVEC may be a useful tool to overcome resistance to checkpoint inhibition [6].

Radiation therapy has been proposed to induce responses to anti-CTLA4 (cytotoxic T-lymphocyte-associated protein) therapy by causing cancer cell lysis. It may increase the expression of tumor-specific antigens in the tumor microenvironment, leading to boosting or priming of effector T cell function against cancer [1]. This putative effect has been previously demonstrated in several cases and case series [3-4, 7].

## Conclusions

In summary, this heavily pre-treated patient who previously progressed on anti-CTLA4, anti-PD1, and TVEC therapies, achieved a complete clinical, histologic and radiographic response after receiving SBRT plus TVEC, followed by ipilimumab. This case allows us to hypothesize that not only the combination of, but also the sequencing of therapies in melanoma may be important by first “priming” and then later “unleashing” the immune system. The case also demonstrates that the lack of response to checkpoint inhibitors may not prohibit reusing them later in the same patient as part of a combination strategy. Therefore, while novel therapies are needed in melanoma, further research into the sequencing and reuse of currently available therapies may also be beneficial. Research in this area could allow us to optimize and obtain the maximum benefit from all available therapies, potentially leading to improved patient outcomes. 
